# Hydralazine and Panobinostat Attenuate Malignant Properties of Prostate Cancer Cell Lines

**DOI:** 10.3390/ph14070670

**Published:** 2021-07-13

**Authors:** Mariana Brütt Pacheco, Vânia Camilo, Nair Lopes, Filipa Moreira-Silva, Margareta P. Correia, Rui Henrique, Carmen Jerónimo

**Affiliations:** 1 Cancer Biology and Epigenetics Group, Research Center of IPO Porto (CI-IPOP)/RISE@CI-IPOP (Health Research Network), Portuguese Oncology Institute of Porto (IPO Porto)/Porto Comprehensive Cancer Center (Porto.CCC), Rua Dr. António Bernardino de Almeida, 4200-072 Porto, Portugal; mariana.brutt.pacheco@ipoporto.min-saude.pt (M.B.P.); vania.gomes.camilo@ipoporto.min-saude.pt (V.C.); nair.ribeiro.lopes@ipoporto.min-saude.pt (N.L.); filipa.m.silva@ipoporto.min-saude.pt (F.M.-S.); margareta.correia@ipoporto.min-saude.pt (M.P.C.); henrique@ipoporto.min-saude.pt (R.H.); 2 Department of Pathology and Molecular Immunology, School of Medicine and Biomedical Sciences, University of Porto (ICBAS-UP), Rua Jorge Viterbo Ferreira 228, 4050-513 Porto, Portugal; 3 Department of Pathology, Portuguese Oncology Institute of Porto (IPOP), Rua Dr. António Bernardino de Almeida, 4200-072 Porto, Portugal

**Keywords:** castration-resistant prostate cancer, epigenetics, HDAC inhibitor, DNMT inhibitor, hydralazine, panobinostat, valproic acid

## Abstract

Among the well-established alterations contributing to prostate cancer (PCa) pathogenesis, epigenetics is an important player in its development and aggressive disease state. Moreover, since no curative therapies are available for advanced stage disease, there is an urgent need for novel therapeutic strategies targeting this subset of patients. Thus, we aimed to evaluate the combined antineoplastic effects of DNA methylation inhibitor hydralazine and histone deacetylase inhibitors panobinostat and valproic acid in several prostate cell lines. The effect of these drugs was assessed in four PCa (LNCaP, 22Rv1, DU145 and PC-3) cell lines, as well as in non-malignant epithelial (RWPE-1) and stromal (WPMY-1) cell lines, using several assays to evaluate cell viability, apoptosis, proliferation, DNA damage and clonogenic potential. We found that exposure to each epidrug separately reduced viability of all PCa cells in a dose-dependent manner and that combined treatments led to synergic growth inhibitory effects, impacting also on colony formation, invasion, apoptotic and proliferation rates. Interestingly, antitumoral effects of combined treatment were particularly expressive in DU145 cells. We concluded that hydralazine and panobinostat attenuate malignant properties of PCa cells, constituting a potential therapeutic tool to counteract PCa progression.

## 1. Introduction

Prostate cancer (PCa) is the second most incident malignancy in men and the fifth leading cause of cancer-related death worldwide [1]. Currently, more than 80% of PCa cases are diagnosed as localized disease [2,3,4], but up to one-third of these patients eventually recur and progress. Due to the important role of androgens and androgen receptor (AR) signaling in normal prostate development and PCa growth [5], the most common treatment for advanced PCa is androgen deprivation therapy (ADT) [3,4]. However, despite initial response to ADT, patients often become refractory and progress to an aggressive disease state, castration-resistant PCa (CRPC), usually within 12 to 30 months [3,6,7]. Although treatment with new generation androgen signaling inhibitors has improved the outcome [8], there are no curative therapies available for these patients [9], entailing the urgent need for novel therapeutic strategies.

The molecular mechanisms underlying progression to CRPC are diverse and complex [5,10]. Similar to other cancer types, epigenetic deregulation was vastly demonstrated as a key factor in PCa development [10,11]. Since DNA methylation can lead to gene silencing, contributing to ADT resistance [12], DNA methyltransferases’ inhibitors (DNMTis) may re-sensitize malignant cells to antineoplastic agents [12,13,14,15]. On the other hand, alterations in particular histone marks also define the epigenetic profile of PCa, consequently altering critical signaling pathways and transcriptional regulation, which contribute to prostate carcinogenesis [5,16]. In particular, histone deacetylases (HDAC) regulate several genes, including AR in prostate cells [17]. Moreover, HDACis hinder histone deacetylation, thus maintaining chromatin structure in a more open conformation, allowing for DNA access and consequent reversion of epigenetically silenced genes by DNMTi. Therefore, HDAC inhibitors (HDACis) are under evaluation in CRPC or chemotherapy-resistant PCa patients due to their effects upon histone modifications. Although the inherent toxicity of DNMTis and HDACis in clinical trials did not support the use of these drugs as single agents for CRPC treatment, reports in pancreatic, lung and breast cancer models suggest that concomitant DNMTi and HDACi treatment is more effective than using each epidrug separately [18,19,20]. Furthermore, these studies suggest that low doses of epigenetic drugs can reprogram tumor cells, re-sensitizing them to conventional therapies [21].

To date, the Food and Drug Administration (FDA) has approved two epigenetic drugs targeting DNMT [22,23,24,25] and four targeting HDAC [26,27,28,29]. Recently, a combination of a DNMTi, 5-aza-2′-deoxycytidine (5-Aza-CdR) and a cytidine deaminase inhibitor, cedazuridine, has been approved for the treatment of myelodysplastic syndromes and chronic myelomonocytic leukemia [30]. Although DNMTis and HDACis have only been approved for hematological malignancies, there are several ongoing clinical trials in solid tumors [3,30,31]. Both FDA-approved DNMT inhibitors, 5-azacytidine (5-Aza-CR) and 5-Aza-CdR, are nucleoside analogues, which are incorporated into the DNA and exert their demethylating action by covalently sequestering DNMTs. However, since this incorporation relies on DNA replication, their action is more pronounced in proliferative cancers than in more indolent ones. PCa generally falls into the second category, which might explain the lack of significant patient benefit in clinical trials using 5-Aza-CR for CRPC [32].

Hydralazine hydrochloride, an FDA-approved drug for treatment of severe hypertension and heart failure [3,31] has been tested as a repurposed drug for cancer treatment. [33]. Several in vitro studies demonstrated that hydralazine displays DNMTi properties and may restore the expression of tumor suppressor genes (TSG) silenced by promoter hypermethylation, without significant cytotoxic effects both in cancer cell lines and primary tumors [33,34,35,36,37]. Importantly, hydralazine is a non-nucleoside analog, directly targeting the catalytic region of DNMT1A and DNMT3A/3B, without being incorporated into DNA [3,38]. Hydralazine efficacy was already tested in clinical trials targeting solid tumors, including cervical [39,40,41,42,43], breast [40,44], lung and ovarian [40] cancer. However, most of the outcomes of these clinical trials remain unreported to date. Almost all of the previously mentioned clinical trials used hydralazine in combination with valproic acid, a therapeutic approach that increased the efficacy of therapy in vitro and caused TSG reactivation [39,40,44]. Valproic acid, a short chain fatty acid inhibitor, is an approved antiepileptic drug, also used for bipolar disorder [43,45]. Furthermore, it has been shown to modulate key pathways—namely, cell cycle arrest, apoptosis, angiogenesis and senescence—through HDAC inhibition both in in vitro and in vivo PCa models [46,47]. Panobinostat is a pan-HDACi approved for treatment of multiple myeloma [48] with demonstrated anti-tumor efficacy in different cell lines and xenograft models [49,50]. The ability to use panobinostat at low and achievable concentrations makes it an attractive candidate for complementary combination with other anti-tumor agents.

Hence, since DNMTs are known to be upregulated in PCa, we hypothesized that epidrugs might be an effective strategy to treat patients with advanced PCa. Our work focused on the DNMTi hydralazine (Hydra), and on the HDACis panobinostat (Pano) and valproic acid (VPA). Thus, we aimed to assess the therapeutic potential of these epigenetic drugs alone or in a combination treatment modality in PCa cell lines. To the best of our knowledge, this is the first study focusing on the effects of the combination of hydralazine and panobinostat in human PCa cells.

## 2. Results

### 2.1. Hydralazine, Panobinostat and Valproic Acid Inhibit PCa Cell Lines Growth

**Figure 1:**
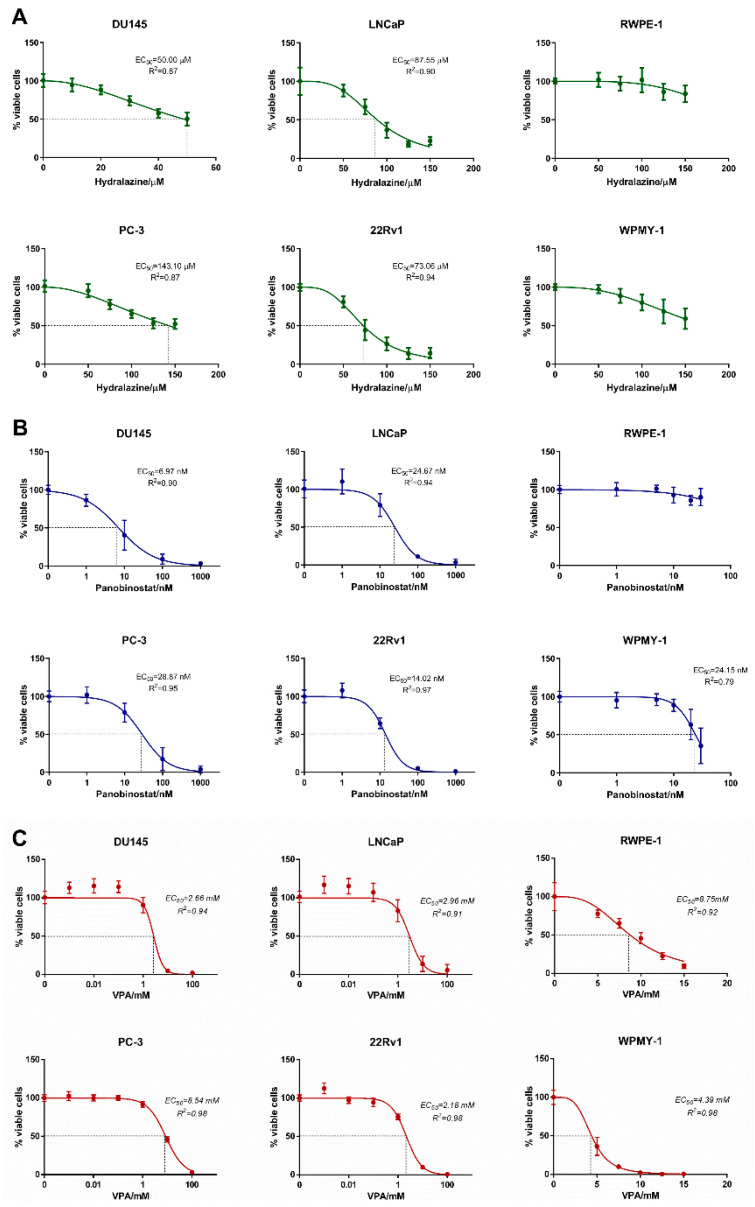
Dose–response curves. Cell viability measured by resazurin in DU145, PC-3, LNCaP, 22Rv1, RWPE-1 and WPMY-1 exposed to hydralazine (**A**), panobinostat (**B**) and valproic acid (**C**) at day 3. Experiments were performed in triplicates of three biological replicates (*n* = 3), normalized to the respective vehicle and to day 0. EC_50_ values at day 3 are indicated in each graph.

We first investigated the effect of each epidrug individually on PCa cell lines viability to determine their half maximal effective concentration (EC_50_) using GraphPad Prism version 6.0. For that, four PCa cell lines (DU145, PC-3, LNCaP and 22Rv1) and two non-malignant prostate cell lines (RWPE-1 and WPMY-1) were treated for 3 days with different concentrations of hydralazine, panobinostat or valproic acid. Among the four PCa cell lines, DU145 was the most sensitive to hydralazine with an EC_50_ of 50.00 µM, followed by 22Rv1 and LNCaP. Conversely, PC-3 was the least responsive PCa cell line, showing similar results to non-malignant WPMY-1 cells. Although the EC_50_ of both non-malignant prostate cell lines could not be determined, RWPE-1 showed to be the most resistant cell line (Figure 1A). Regarding panobinostat, the response window was in the range of nM in all cell lines. Once more, DU145 was the most sensitive, with an EC_50_ of 6.97 nM, followed by 22Rv1, LNCaP and PC-3. Although its EC_50_ could not be determined, RWPE-1 was the least sensitive cell line of all tested, whereas WPMY-1 disclosed an EC_50_ of 24.15 nM (Figure 1B). DU145, 22Rv1 and LNCaP showed similar response to valproic acid treatment, with EC_50_ of approximately 2–3 mM, while PC-3 was the least responsive. Non-malignant cell lines WPMY-1 and RWPE-1 exhibited EC_50_ of 4.46 mM and 8.97 mM, respectively (Figure 1C). Overall, DU145 was the most sensitive cell line to all drugs individually, whereas PC-3 was the least responsive cancer cell line. In general, the EC_50_ values observed for the non-malignant cell lines were higher than those of PCa cell lines, indicating that exposure of these cells to the concentrations required for counteracting malignant cells is likely to pose no significant toxicity to normal cells.

### 2.2. Hydralazine and Panobinostat Synergize in Inhibiting Cell Growth in PCa Cell Lines

**Figure 2:**
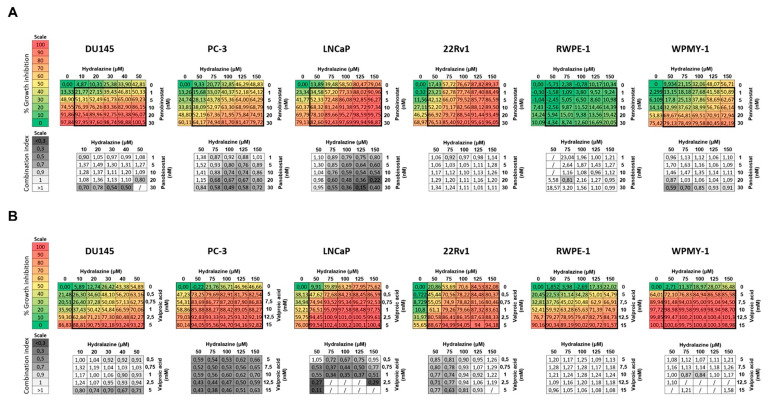
Combination matrix. Cells treated with hydralazine and panobinostat (**A**) or valproic acid (**B**) at the indicated concentrations for three days. Each plate contained 6 × 6 dose matrix blocks with several concentrations of both drugs. Percentage of growth inhibition was calculated for each epidrug combination using resazurin and are representative of three biological replicates (*n* = 3). The combination index (CI) was calculated using CompuSyn software, where <1 indicates synergism, =1 is additive effect and >1 indicates antagonism. The percentage and color codes for growth inhibition and CI scales are shown on the left. The results that CompuSyn could not convert into CI, since the effect values were not between 0 and 1, are represented by /.

Next, we sought to ascertain whether the treatment of PCa cell lines with both hydralazine and panobinostat or valproic acid resulted in synergistic cell growth inhibition. Thus, we combined both drugs in a 6 × 6 checkerboard system using the respective EC_50_ values as a starting point. The combination index (CI) was assessed to determine whether the combination was synergistic (<1), additive (=1) or antagonistic (>1). Synergy between hydralazine and panobinostat was observed in DU145, PC-3 and LNCaP, mostly at higher concentrations (Figure 2A). The CI value obtained for DU145 cells treated with 50 µM hydralazine and 20 nM panobinostat was 0.80, whereas for PC-3 and LNCaP cell lines, CI values of 0.74 and 0.59 were obtained following treatment with 100 µM hydralazine and 10 nM panobinostat. Hence, hydralazine and panobinostat synergized in these cell lines as cell growth inhibition was attained at these concentration ranges. Moreover, these epidrug combinations did not severely impact on the growth of non-malignant cell lines, synergism only being observed at higher panobinostat concentrations (≥20 nM). Likewise, hydralazine and valproic acid demonstrated a synergistic effect in all PCa cell lines (Figure 2B). However, the effective range of concentrations was several orders of magnitude higher for VPA (mM) compared with panobinostat (nM). Thus, considering that panobinostat induced synergist effects similar to VPA at much lower doses, we focused on the combination of hydralazine with panobinostat, which was unexploited, thus far.

To proceed for functional assays, we selected the best hydralazine–panobinostat combination for each cell line, considering EC_50_ and CI values. Although, in DU145 cells, synergism was mostly observed at higher panobinostat levels (higher than respective EC_50_), a lower concentration was used to avoid toxicity (Table 1).

### 2.3. Hydralazine and Panobinostat Induce Apoptosis and DNA Damage in PCa Cell Lines

**Figure 3:**
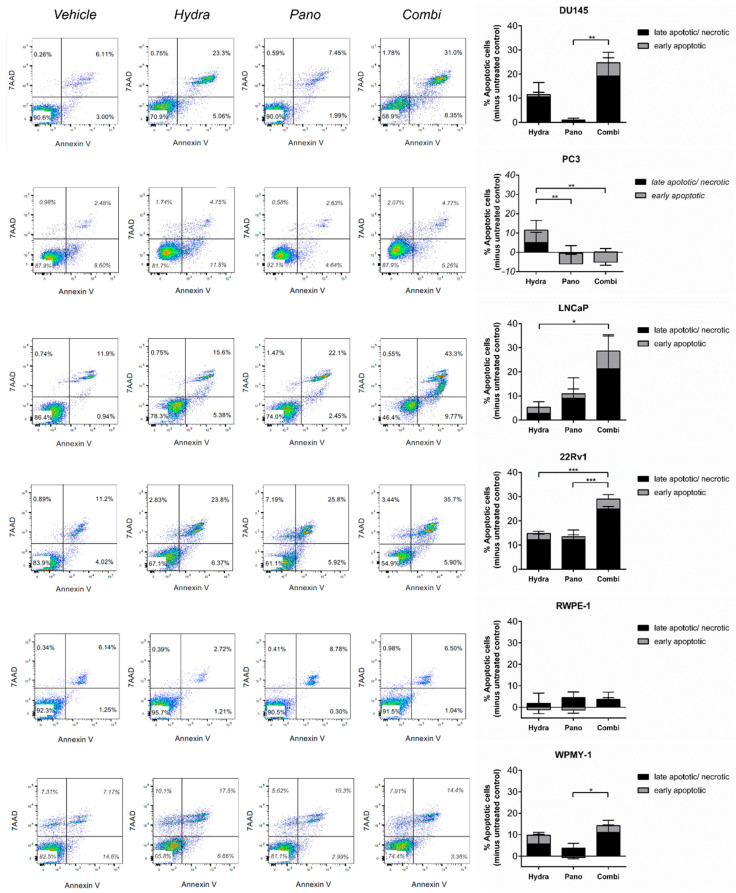
Hydralazine and panobinostat exposure induced apoptosis in DU145, LNCaP and 22Rv1. Cells were treated with the selected combinations, stained with annexin V and 7-AAD and analyzed by flow cytometry. A representative image is shown (**left panel**). The percentage of apoptotic cells of DU145, LNCaP and 22Rv1 is shown in the right panel. Experiments were carried out in three biological replicates (*n* = 3). Mean values ± SD are shown (**right panel**), analyzed by two-way ANOVA with Tukey’s test for multiple comparisons. Drug concentrations: DU145–Hydra: 50 µM, Pano: 5 nM; PC-3, RWPE-1 and WPMY-1–Hydra: 100 µM, Pano: 10 nM; LNCaP–Hydra: 100 µM, Pano: 5 nM; 22Rv1–Hydra: 75 µM, Pano: 1 nM). * *p* < 0.05; ** *p* < 0.01; *** *p* < 0.001.

To assess if the previously observed growth inhibition was due to apoptosis induction, the percentage of apoptotic and necrotic cells were assessed in PCa cell lines treated with hydralazine and panobinostat, alone or in combination. The combined treatment led to an increased percentage of apoptotic/necrotic cells compared with each individual treatment alone (Figure 3). Contrarily, PC-3 did not respond to either treatment modality. As expected, no effect was apparent in the non-malignant cell lines. The effect of treatment with hydralazine, panobinostat and the combination approach on the number of viable cells at day 3 is visible in Figure S1A. 

**Figure 4:**
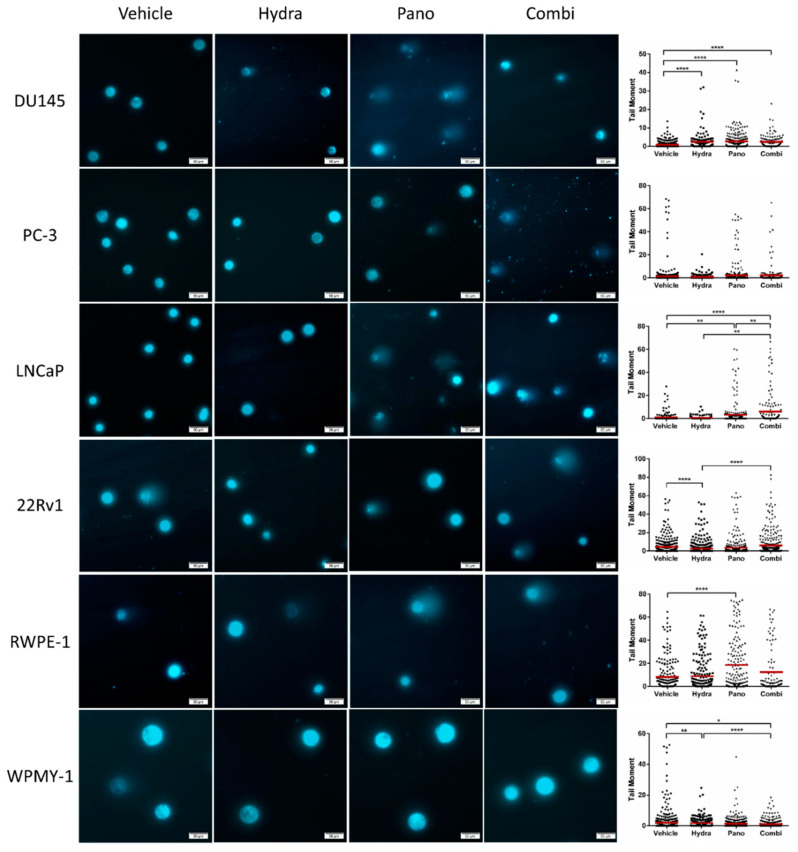
Effect of combined hydralazine and panobinostat treatment in DNA damage of PCa cell lines by comet assay. DNA damage evaluation was determined by measuring tail moment (tail % DNA × means of head × tail distance) of at least 50 comets per condition. Representative pictures were taken with Olympus IX51 microscope at 200 × magnification (scale bar 50 μm) (**left panel**). Results are represented by the mean of three biological replicates (*n* = 3) analyzed by Kruskal–Wallis with Dunn’s test for multiple comparisons (**right panel**). Drug concentrations: DU145–Hydra: 50 µM, Pano: 5 nM; PC-3, RWPE-1 and WPMY-1–Hydra: 100 µM, Pano: 10 nM; LNCaP–Hydra: 100 µM, Pano: 5 nM; 22Rv1–Hydra: 75 µM, Pano: 1 nM). * *p* < 0.05; ** *p* < 0.01; **** *p* < 0.0001.

As both hydralazine and panobinostat *per se* have been linked to DNA damage, the effect of combined treatment on DNA damage was also evaluated using the alkaline comet assay. Remarkably, global DNA fragmentation was significantly higher after the combined treatment in LNCaP compared with each single treatment alone, whereas in 22Rv1, differences were only apparent for the condition with hydralazine alone (Figure 4). Surprisingly, though both hydralazine and panobinostat increased DNA damage individually, their combination did not result in additional DNA fragmentation in DU145 cells. In accordance with apoptosis results, both treatment modalities did not increase DNA damage in PC-3 and WPMY-1 cells. Nevertheless, panobinostat significantly increased tail moment in RWPE-1 cells.

### 2.4. Proliferation and Colony Formation Decreased in DU145 after Combined Treatment

**Figure 5:**
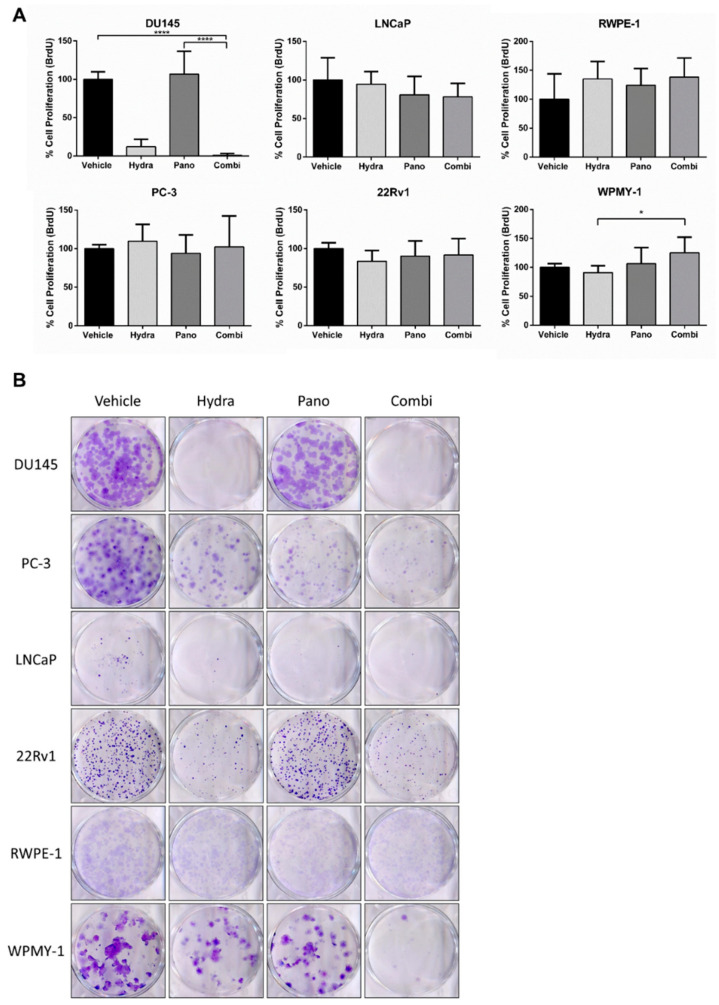
Effect of combined epidrug treatment on cell proliferation and colony formation. (**A**) The percentage of cell proliferation was determined by BrdU assay at day 3 of treatment with hydralazine and/or panobinostat. Experiments were performed in triplicates (*n* = 3), normalized to the respective vehicle and to day 0. Mean values ± SD are shown, analyzed by Kruskal–Wallis with Dunn’s test for multiple comparisons. (**B**) Representative images of the colony formation assay performed in prostate cell lines exposed to hydralazine and/or panobinostat for 3 days. Colony formation was followed for 7 additional days (10 days for LNCaP). Experiments were carried out in triplicates (*n* = 3). The effect was compared with the vehicle condition. Drug concentrations: DU145–Hydra: 50 µM, Pano: 5 nM; PC-3, RWPE-1 and WPMY-1–Hydra: 100 µM, Pano: 10 nM; LNCaP–Hydra: 100 µM, Pano: 5 nM; 22Rv1–Hydra: 75 µM, Pano: 1 nM). * *p* < 0.05; **** *p* < 0.0001.

Subsequently, the impact of the combined epidrug treatment on cell proliferation was also evaluated based on BrdU incorporation. Only DU145 cells’ proliferation was affected by the treatment with these drugs (Figure 5A). Specifically, treatment with hydralazine alone, but not panobinostat, led to considerable proliferation inhibition compared with the vehicle. Nevertheless, although without any statistically significant differences between hydralazine and the combination approach, this inhibitory effect was slightly increased by combined treatment (Figure 5A). The alterations observed in LNCaP cells were less striking, but proliferation tended to decrease slightly with treatment, especially with the combined approach (Figure 5A). Similarly, the clonogenic capacity of DU145 single cells after treatment exposure was dramatically reduced, as almost no colonies were apparent after exposure to hydralazine or to combined treatment (Figure 5B). Conversely, treatment with panobinostat alone showed a higher impact on PC-3 and LNCaP cell lines. Furthermore, the combined treatment only affected colony formation ability in PC-3. RWPE-1 and WPMY-1 cells were not affected by any condition, with the exception of the combined treatment in WPMY-1, after which only a reduced number of colonies were formed (Figure 5B).

### 2.5. DU145 Cell Invasion and Migration Was Decreased with Combined Treatment

**Figure 6:**
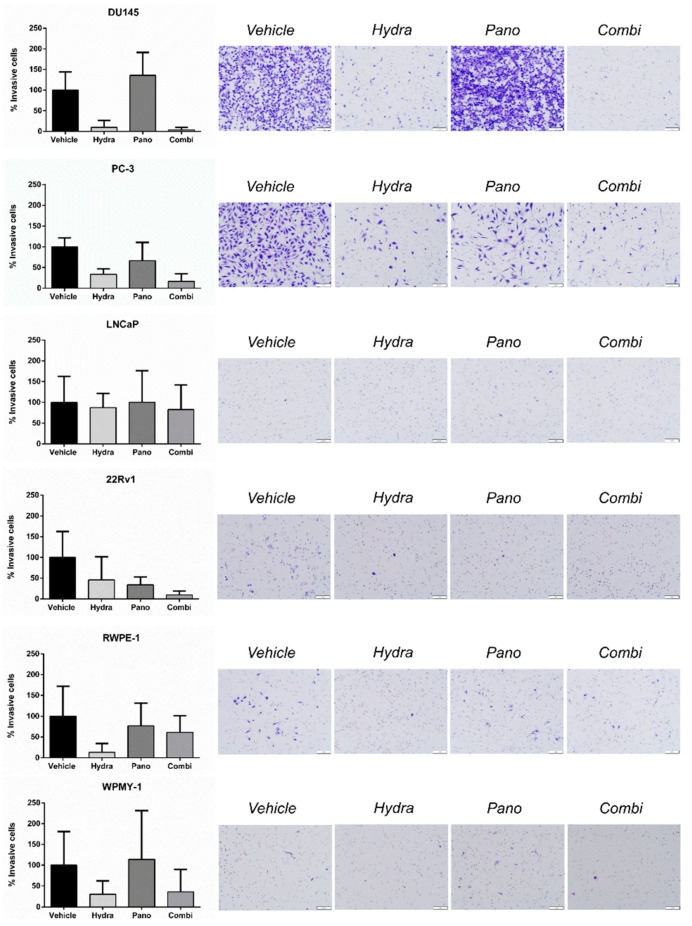
Effects of hydralazine and panobinostat exposure in prostate cell lines migration potential. Cell lines were seeded in migration invasion transwell inserts upon epidrug exposure for 24 h. The percentage of invasive and migratory cells was determined by dividing the mean counted cells by the area of the microscope viewing field and then multiplying by the entire area of the insert. The results are representative of three biological replicates (*n* = 3). Mean values ± SD are shown (**left panel**). Representative images of invasive and migratory cells stained with Crystal Violet are displayed (**right panel**). The inserts were photographed with stereomicroscope Olympus S2X16 using a digital camera Olympus SC180 (scale bar 1 mm) at 115 × magnification. Drug concentrations: DU145–Hydra: 50 µM, Pano: 5 nM; PC-3, RWPE-1 and WPMY-1–Hydra: 100 µM, Pano: 10 nM; LNCaP–Hydra: 100 µM, Pano: 5 nM; 22Rv1–Hydra: 75 µM, Pano: 1 nM).

In parallel, the effect of the epidrugs in PCa cell lines’ invasive capabilities was tested using an invasion transwell assay. Twenty-four hours after seeding, the vehicle-treated cell lines with the highest percentage of invasive cells were DU145 and PC-3, whereas for LNCaP and 22Rv1 this percentage was only marginal (below 0.3%) (Figure 6). Nevertheless, 22Rv1 cells’ invasion reduction upon treatment with each epidrug individually was further enhanced upon combination. Moreover, DU145 cells’ exposure to hydralazine alone, but not panobinostat, also reduced invasion, the effect of combined treatment being only slightly superior to hydralazine’s effect. Likewise, the effect of hydralazine upon PC-3 invasive capabilities was two times higher than that of panobinostat, the combined treatment being able to further reduce the percentage of invasive cells. Nevertheless, these differences were not statistically significant (Figure 6).

**Figure 7:**
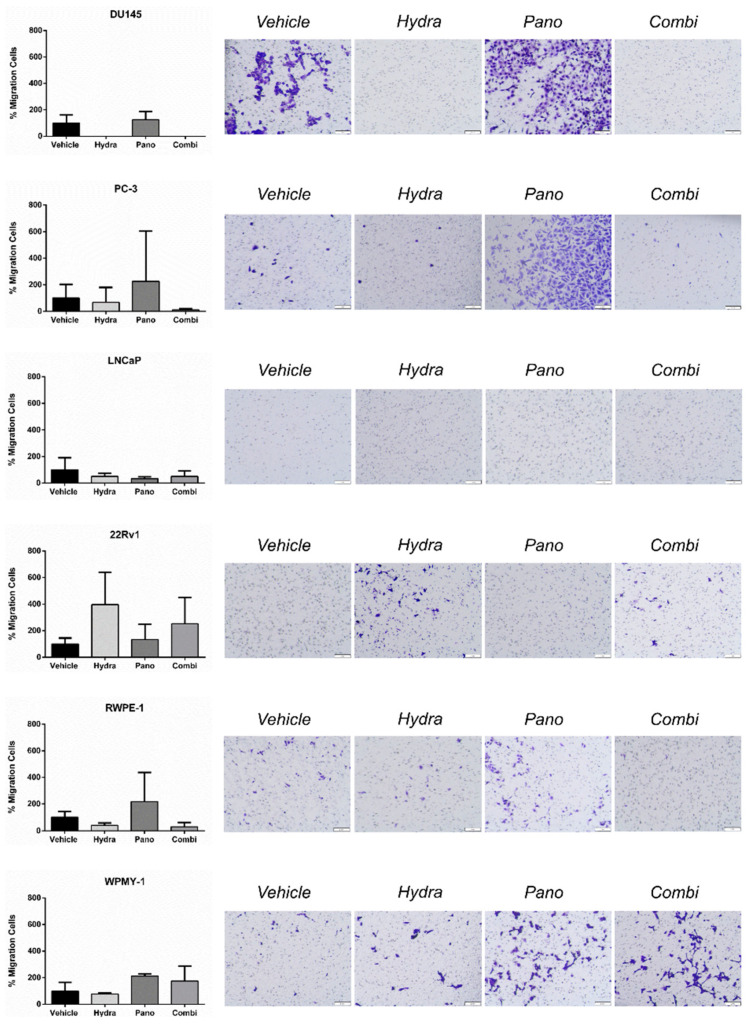
Effects of hydralazine and panobinostat exposure in prostate cell lines migration potential. Cell lines were seeded in migration invasion transwell inserts upon epidrug exposure for 24 h. The percentage of invasive and migratory cells was determined by dividing the mean counted cells by the area of the microscope viewing field and then multiplying by the entire area of the insert. The results are representative of three biological replicates (*n* = 3). Mean values ± SD are shown (left panel). Representative images of invasive and migratory cells stained with Crystal Violet are displayed (right panel). The inserts were photographed with stereomicroscope Olympus S2X16 using a digital camera Olympus SC180 (scale bar 1 mm) at 115 × magnification. Drug concentrations: DU145–Hydra: 50 µM, Pano: 5 nM; PC-3, RWPE-1 and WPMY-1–Hydra: 100 µM, Pano: 10 nM; LNCaP–Hydra: 100 µM, Pano: 5 nM; 22Rv1–Hydra: 75 µM, Pano: 1 nM).

Additionally, the migration capabilities of PCa cell lines were evaluated through a migration transwell assay. In accordance with the invasion assay, but to a lesser extent, the vehicle-treated DU145 cells displayed the highest percentage of migratory cells compared with the other cell lines. Furthermore, DU145 cells’ exposure to hydralazine alone or in combination, but not to panobinostat alone, reduced cell migration (Figure 7). However, the percentage of migratory cells for the remainder of the untreated PCa cell lines was quite low (below 0.5%). Nonetheless, no significant differences were observed between the different treatment modalities (Figure 7). 

## 3. Discussion

Prostate cancer is one of the most common and lethal malignancies among men, worldwide [1]. Most PCa patients display androgen-dependent tumors at diagnosis, but a sizeable proportion progresses to CRPC following ADT [2,3,4]. At this stage, further therapeutic interventions are of limited success, mostly offering symptomatic relief, increasing patients’ quality of life but with little effect on survival. Thus, despite the multiple approved therapies, CRPC remains an incurable disease [8,9]. Since epigenetic changes, particularly aberrant gene promoter hypermethylation and histone deacetylation, are frequent events in the molecular pathogenesis of PCa [10], we hypothesized that epidrugs might constitute a promising treatment strategy for advanced PCa. 

Hence, the aim of this study was to evaluate whether the combination of DNMTi hydralazine and HDACi panobinostat or VPA would be advantageous in comparison with their use as single therapeutic agents in PCa. Although the combination of epidrugs with demethylating and deacetylating properties is increasingly common, to our knowledge this is the first report that addresses the combination of hydralazine and panobinostat in PCa. Thus, we selected two metastatic CRPC cell lines, DU145 and PC-3 (both AR−), and two metastatic, androgen-sensitive PCa cell lines, LNCaP and 22Rv1. Additionally, Chou–Talalay method was applied through CompuSyn to assess synergism between two drugs for the selection of the optimal concentrations to be tested in the functional assays. Nevertheless, hydralazine and panobinostat synergized only at higher panobinostat concentrations in the DU145 cell line. Thus, since the goal was to cause non-cytotoxic effects with this combination, and since panobinostat might potentiate hydralazine in DU145 cell line, a lower panobinostat concentration was used in all functional assays. However, the CI value for this selected concentration was 1.27, indicating that this combination might be between the additive and antagonistic effect. Notwithstanding, and as previously reported, defining “additive effect” has been the most challenging criterion for distinguishing synergism from antagonism [51]. In this context, the *Highest Single Agent* (HSA) approach, which reflects a higher resulting effect of a drug combination than the effects produced by its individual components, is the most appropriate [52]. As the hydralazine–panobinostat combination effect (69.23% growth inhibition) was higher than each epidrug individually (hydralazine—42.81% and panobinostat—48.90%, growth inhibition; Figure 2A), the HSA approach supports the panobinostat concentration utilized in the additional assays.

In accordance with previously published data from our team [3], in this study we confirmed that hydralazine treatment alone impairs the neoplastic capabilities of different PCa cell lines. We have also shown here, for the first time, that non-malignant prostate cell lines, representative of the epithelial and stromal prostate tissue withstand higher doses of hydralazine than their malignant counterparts. This is also true for panobinostat and VPA, for all the PCa cell lines tested, except PC-3. 

This is an important finding since antitumoral therapeutic approaches clearly benefit from the use of epidrug concentrations that cause minimal cytotoxic damage to adjacent normal tissue.

For single treatment regimens, we observed that all PCa cell lines were sensitive to the effects of these drugs, albeit at different levels. Among all PCa cell lines tested, and regardless of the epidrug that was used, DU145 was the most responsive and PC-3 was the least sensitive. Differential epidrugs sensitivity and underlying mechanisms have not yet been investigated in these two PCa cell lines. However, it is acknowledged that PC-3 is more aggressive than DU145, reflecting their metastatic sites, which are bone and brain, respectively. We hypothesize that DU145 might display more epigenetic aberrations and thus might be more responsive to epidrugs than PC-3. For many of the tested concentrations, the combination of hydralazine with panobinostat (or VPA) had a superior effect on growth inhibition than individual treatment and, at specific epidrug concentrations, a synergistic effect was observed. The mechanisms by which these drugs led to a hampered growth were also cell-line dependent, but in most of the cases this inhibitory effect could be linked to either increased apoptosis or to a proliferation impediment, which may be partially attributed to the induction of DNA damage following treatment. It is important to highlight that although DNA damage was also observed for the non-malignant cell line RWPE-1 following panobinostat treatment, unlike cancer cells, non-malignant cells seem to be able to repair and recover from HDACi-induced damage after epidrug removal. Similarly, the WPMY-1 cell line might also recover from the slightly increased rate of apoptosis in the combination approach after epidrug removal [53]. In accordance, for the non-malignant cell line RWPE-1, one week after treatment cessation, no significant differences in the number of colonies between vehicle and panobinostat-treated cells were apparent. Contrarily, for WPMY-1, there was a decrease in colonies number in the combined approach.

Finally, for metastatic CRPC cell lines DU145 and PC-3, treatment negatively affected the invasion potential. While this result is noteworthy in terms of further dissemination of CRPC, it would be important to assess whether these treatments would also limit metastatic spread of non-metastatic CRPC tumors.

Although HDACis are actively explored as new-generation epidrugs, they have low efficacy in cancer monotherapy, especially in solid tumors [54]. Moreover, HDACis might induce multiple cytotoxic actions in cancer cells. For instance, pro-invasive effects have been reported in melanoma cells *in vitro*, due to upregulation of N-cadherin expression and RhoA activity inhibition [55]. Additionally, cell migration activity as well as metastasis formation were dramatically enhanced by various classes of HDACi treatment in human breast, gastric, liver and lung cancer cell lines [54]. Likewise, we observed that single panobinostat treatment led to increased cell migration and invasion in some of the PCa cell lines. Hence, this pan-HDACi might be inducing pro-invasive/migratory behavior. Interestingly, when hydralazine was added, this enhancement was not observed. These findings warrant caution regarding the single use of these agents in cancer treatment and in their possible resistance mechanisms. Indeed, several studies supported that HDACis work synergistically with a range of diverse chemical compounds [56,57,58].

Previous reports from our group [3] and others [59,60,61] had already demonstrated that hydralazine and panobinostat were DNA damage-inducing agents. The mechanisms underlying the effect of panobinostat can be traced to its capacity to modulate DNA damage response (DDR) proteins [62,63] and to the induction of reactive oxygen species (ROS) [64,65], a characteristic that is shared with other HDACis [66,67]. These features suggest its use in combination with conventional radiotherapy. In fact, in vitro evidence in prostate [62] and bladder [63] cancer models supports panobinostat as a radiosensitizer. For hydralazine, the mechanistic dissection of these effects is still largely undisclosed, but it may also be associated with the modulation of DDR proteins since previous studies showed that exposure to hydralazine impacted PARP1, RAD51 and WAF1 expression levels [3]. Additionally, it may also be associated with the generation of ROS species, as observed in a T cell leukemia model [59]. In support of this, DNMTi zebularine and decitabine apoptosis-inducing effects, mainly attributed to DNA damage, were also intrinsically associated with ROS production [68]. Interestingly, decitabine and zebularine have also been proposed to act as radiosensitizers through yet unidentified mechanisms [69]. Although these observations warrant further investigation, one may hypothesize that combining hydralazine (alone or with HDACis) to radiotherapy for PCa management may be of therapeutic value.

Contrary to hydralazine, the benefit of panobinostat as a therapeutic agent against PCa has already been addressed in clinical trials. In a phase II clinical trial, panobinostat failed to demonstrate significant clinical efficacy as a single agent in CRPC patients with disease progression following chemotherapy (NCT00667862) [70]. However, its combination with antiandrogen bicalutamide in a phase I/II trial led to delayed radiographic progression, suggesting a clinical benefit (NCT00878436) [71]. The rationale for using this combination comes from in vitro evidence showing HDACi modulation of both AR expression and its transactivation capacity [72]. Furthermore, as AR directly and indirectly regulates several DDR genes, AR+ cells would be more susceptible to the effect of panobinostat. Accordingly, in our study, apoptosis following treatment with panobinostat alone was more pronounced in AR+ cells than in AR− cells. Importantly, these AR+ cells benefited from a combination approach with hydralazine. Conversely, in the AR− DU145 cell line, the effects observed were mainly attributable to hydralazine, with panobinostat alone showing meagre effects. We are tempted to speculate that this might be related to hydralazine’s regulation of signaling networks that are essential for DU145 cells in the absence of AR. 

Overall, our results suggest a therapeutic benefit in the combination of hydralazine and panobinostat for PCa management, with limited toxicity and good tolerance by non-malignant epithelial and stromal prostate cells. In fact, our concentrations are physiologically relevant since they do not exceed the already approved ones. The maximum clinically recommended dose of hydralazine is 200 mg/day, and its bioavailability is 16% for fast acetylators and 35% for slow ones [73]. Thus, this broadly corresponds to a concentration of 0.2 M and 0.44 M for fast and slow acetylators, respectively. Regarding panobinostat, the maximum clinical dose is 30 mg, meaning that with a bioavailability of 21.4% it corresponds approximately to a concentration of 18.40 mM [74]. Considering that the maximal concentrations used in our study (100 µM for hydralazine and 10 nM for panobinostat) are significantly lower than the ones used in the clinic, they should be considered clinically relevant. In vivo studies are now mandatory to assess more specifically the effectiveness of this combination treatment and, importantly, its toxicity, to further proceed to efficacy and safety evaluation in clinical trials.

## 4. Materials and Methods

### 4.1. Cell Lines

Human epithelial PCa cell lines (DU145, LNCaP, 22Rv1 and PC-3) as well as non-malignant epithelial and stromal prostate cell lines (RWPE-1 and WPMY-1, respectively) available at our laboratory were maintained at 37 °C in a humidified atmosphere containing 5% CO_2_ in low passages and grown in adequate media. Specifically, DU145 cells were maintained in MEM (Biotecnómica, Porto, Portugal), whereas PC-3, LNCaP, 22Rv1 and WPMY-1 were grown in RPMI (Biotecnómica, Porto, Portugal). RWPE-1 was cultured in K-SFM (Biotecnómica, Porto, Portugal). All media were supplemented with 10% fetal bovine serum (FBS, Biochrom, Merck, Darmstadt, Germany) and 1% penicillin–streptomycin (GIBCO, Invitrogen, Waltham, MA, USA). All prostate cell lines were routinely tested for *Mycoplasma* spp. contamination using two primers: GP01: ACTCCTACGGGAGGCAGCAGTA and MGS0: TGCACCATGTGTCACTCTGTTAACCTC (Sigma-Aldrich, St. Louis, MO, USA).

### 4.2. Drugs

Hydralazine hydrochloride (Tokyo Chemical Industry, Oxford, UK) was freshly dissolved in sterile distilled water (dH_2_O) (B. Braun, Melsungen, Germany) to the concentration of 0.1 M each day of treatment. Panobinostat (Selleck Chemicals, Houston, TX, USA) was diluted to a 1 μM concentration. Valproic acid (Tokyo Chemical Industry, Oxford, UK) was dissolved in culture medium at 100 mM at the beginning of each experiment.

### 4.3. Cell Viability Assay

Briefly, PCa cell lines were seeded into 96-well plates at 5000 cells per well and incubated overnight at 37 °C in 5% CO_2_. Subsequently, cells were treated with a range of concentrations of each epidrug and their respective vehicle (dH_2_O for hydralazine; dimethyl sulfoxide (DMSO, Sigma-Aldrich, St. Louis, MO, USA) for panobinostat and culture medium for valproic acid) during three consecutive days, exchanging the medium every 24 h. Three experimental replicates of three biological replicates were used for each assay. 

The cell viability was evaluated using resazurin (Canvax Biotech, Córdoba, Spain) at 0 h and 72 h of treatment. The culture medium was removed, and cells were incubated for three hours in the dark at 37 °C and 5% CO_2_ with 100 μL of 1:10 resazurin solution in culture medium. Then, the solution was removed, and the absorbance was measured using a microplate reader (FLUOstar Omega, BMG Labtech, Ortenberg, Germany) at a wavelength of 560 nm with background subtraction at 600 nm. The OD values were corrected using resazurin solution as blank.

### 4.4. Synergy Assay

Cells were seeded in 96-well plates and treated the following day with several drug combinations in a checkerboard system. Each plate contained 6 × 6 dose matrix blocks with several epidrug concentrations below and above each respective EC_50_ value. Additional wells were reserved for untreated and vehicle-treated control wells. Twenty-four hours after the last treatment, the percentage of growth inhibition was assessed using resazurin assay. The combination index (CI) was calculated using CompuSyn software, where <1 indicates synergism, =1 is an additive effect and >1 indicates antagonism. This calculation does not invoke any statistical principles, methods or assumptions. Instead, it is derived via mathematical induction and deduction of several hundred derived equations from enzyme kinetic models with different reaction mechanisms in the presence of an inhibitor. Interestingly, this derived general theory of dose and effect has been demonstrated to be the unified theory of four basic equations: Henderson–Hasselbalch, Michaelis–Menten, Hill and Scatchard equations [51]. 

All experiments were performed with biological triplicates.

### 4.5. Proliferation Assay

The cell proliferation ELISA BrdU (5-bromo-2′ deoxyuridine) assay (Roche Applied Sciences, Germany) was performed at 72 h of treatment. Cells were plated into 96-well plates at a density of 5000 cells per well, and at 0 h and 72 h of treatment, cells were incubated with 10 µM BrdU labeling solution for 8 h. After removing the culture medium, the cells were fixed with FixDenat solution for 30 min at room temperature. Then, the anti-BrdU-POD antibody (1:100) was added. After three washes with PBS 1 ×, the immune complexes were detected by the subsequent substrate reaction. The reaction product was quantified in a microplate reader (FLUOstar Omega, BMG Labtech, Ortenberg, Germany) at a wavelength of 450 nm with background subtraction at 690 nm. All ODs were normalized for the 0 h time point as well as to the vehicle. Three biological and three experimental replicates were used for each condition. 

### 4.6. Apoptosis Assay

Apoptosis was assessed using FITC Annexin V Apoptosis Detection Kit with 7-aminoactinomycin D (7-AAD) (Biolegend, Dedham, MA, USA), according to manufacturer’s instructions. Briefly, a total of 1 × 10^5^ cells were seeded in 6-well plates and treated for three days. Then, cells were harvested and washed twice with cold cell staining buffer (Biolegend, Dedham, MA, USA). Afterwards, cells were resuspended in Annexin V Binding buffer and stained with FITC Annexin V and 7-AAD for 15 min in the dark at room temperature. Cells were acquired in flow cytometer (FACS Canto^TM^ II Cell Analyzer, BD Bioscience, Franklin Lakes, NJ, USA) and analyzed using FlowJo^TM^ software. Three biological replicates were used for each condition.

### 4.7. Invasion and Migration Assay

Cell invasion and migration were determined using Falcon^®^ Permeable Support for 24-well Plate with 8.0 µm Transparent PET Membrane (Corning, New York, NY, USA) and Nunc^®^ Cell Culture Inserts in 24-well Nunclon Delta surface plate (Sigma-Aldrich, St. Louis, MO, USA), respectively. Briefly, cells were harvested after treatment and were added to the upper chamber in serum-free medium, according to its optimized density (2.5 × 10^4^ cells for every cell line except from LNCaP and 22Rv1 with 5 × 10^4^ cells). Media containing 10% FBS was added to the lower chamber. After 24 h incubation at 37 °C and 5% CO_2_, the cells remaining on the upper side of the membrane were removed with cotton swabs, and those on the lower surface of the membrane were fixed in methanol (Supelco, Sigma-Aldrich, St. Louis, MO, USA), washed in PBS 1 × and stained with Crystal Violet (Active motif, Carlsbad, CA, USA). All the inserts were photographed with stereomicroscope Olympus S2X16 using a digital camera Olympus SC180 (scale bar 1 mm) at 12.5 × magnification. Five fields within each insert were selected and photographed at 115 × and further counted using ImageJ software Cell Counter Plugin. The mean of the counted cells was calculated, divided by the area of the microscope viewing field and then multiplied by the entire area of the insert. Three biological replicates were used for each condition.

### 4.8. Colony Formation Assay

PCa cells were seeded in 6-well culture plates at specific concentrations for each cell line, as detailed in Table S1. After epidrug exposure, cells were incubated at 37 °C and 5% CO_2_ for 7 days and 10 days (LNCaP). Next, colonies were fixed in methanol (Supelco, Sigma-Aldrich, St. Louis, MO, USA), washed in PBS 1 × and stained with Hemacolor Solution II and III (Sigma-Aldrich, St. Louis, MO, USA). Three biological and three experimental replicates were used for each condition. 

### 4.9. Alkaline Comet Assay

After epidrug exposure, 30,000 cells were harvested by trypsinization, washed in PBS, re-suspended in 0.5% low-melting point agarose (*w*/*v*) and immediately placed on a microscope slide previously covered with 1% normal-melting point agarose (*w*/*v*). Then, the cells were immersed in lysis solution (2.5 M NaCl, 100 mM Na_2_EDTA, 10 mM Tris Base and 1% Triton X-100, pH 10) at 4 °C during 2 h in the dark. Afterwards, the slides were incubated in an alkaline electrophoresis buffer (300 mM NaOH, 1 mM Na_2_EDTA, pH 13) for 40 min at 4 °C to allow DNA unwinding. Single cell gel electrophoresis was performed on a horizontal electrophoresis platform for 30 min at 21 V, 300 mA, 4 °C. Next, slides were incubated in a neutralization buffer (0.4 M Tris-Base, pH 7.5) for 10 min, followed by staining with DAPI. Comet analysis was done using OpenComet v.1.3.1. Global DNA damage (SSB and DSB) evaluation was determined by measuring tail moment (tail % DNA × means of head × tail distance) and representative pictures taken with Olympus IX51 microscope at 200 × magnification (scale bar 50 μm). A sampling of at least 50 comets was included in the analysis. Three biological replicates were used for each condition. 

### 4.10. Statistical Analysis

Non-parametric tests (Kruskal–Wallis) among groups with Dunn’s correction and two-way ANOVA with Tukey’s multiple comparisons test were used to compare different conditions through GraphPad Prism version 6.0. All results are shown as the mean ± SD for each group. For each analysis, *p* values were considered significant when inferior to 0.05 (* *p*  <  0.05; ** *p * <  0.01; *** *p*  <  0.001; **** *p*  <  0.0001). All differences between groups without any indicated asterisk were not statistically significant.

## 5. Conclusions

In summary, we demonstrated that the DNMTi hydralazine in combination with the HDACi panobinostat have synergistic anticancer effects, especially upon DU145, LNCaP and 22Rv1 cell lines. This combination effectively reduces cell viability, cell proliferation and colony formation, while it increases total apoptosis, DNA damage as well as invasion and migration, compared with each drug individually. In vivo studies are now mandatory to further validate this promising combination treatment for PCa patients.

## Figures and Tables

**Table 1:** Selected concentrations utilized in the functional assays. Each combination was selected according to each cell line EC_50_ and the CI values.

	Hydralazine (Hydra)	Panobinostat (Pano)	Combination (Combi)
DU145	50 µM	5 nM	50 + 5
PC-3	100 µM	10 nM	100 + 10
LNCaP	100 µM	5 nM	100 + 5
22Rv1	75 µM	1 nM	75 + 1
RWPE-1	100 µM	10 nM	100 + 10
WPMY-1	100 µM	10 nM	100 + 10

## Data Availability

The main data supporting the findings of this study are available within the paper and its Supplementary Materials.

## Supplementary Materials

The following are available online at https://www.mdpi.com/article/10.3390/ph14070670/s1, Table S1: Number of cells seeded in 6-well culture plates for the Colony Formation Assay, Figure S1: Number of viable cells at day 3 of treatment with hydralazine, panobinostat and the combination treatment. Cells were treated with the selected combinations, harvested after treatment and counted. The seeded cells (1 × 10^5^) were subtracted from the number of viable cells of every cell line. Experiments were carried out in three biological replicates (*n* = 3).
