# Supplementary material for: Hydralazine and Panobinostat Attenuate Malignant Properties of Prostate Cancer Cell Lines
Source: Pharmaceuticals (Basel). 2021 Jul 13;14(7):670. doi: 10.3390/ph14070670 (PMC8308508; doi:10.3390/ph14070670)
Supplement: Supplementary file 1 [file pharmaceuticals-14-00670-s001.zip › pharmaceuticals-1245591-supplementary.pdf]

Table S1. *Number of cells seeded in 6-well culture plates for the Colony Formation Assay.*

	**No. of seeded cells**
**DU145**	750
**PC-3**	750
**LNCaP**	1500
**22Rv1**	1125
**RWPE-1**	1500
**WPMY-1**	750

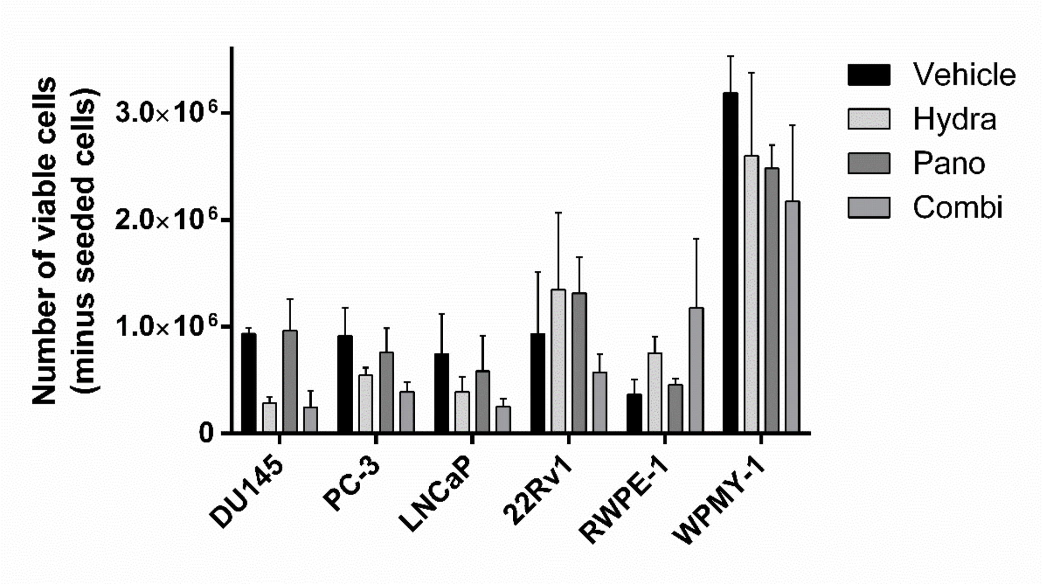

Figure S1. Number of viable cells at day 3 of treatment with Hydralazine, Panobinostat and the combination treatment. *Cells were treated with the selected combinations, harvested after treatment and counted. The seeded cells (1 x 10^5) were subtracted to the number of viable cells of every cell line. Experiments were carried out in three biological replicates (n=3).*
